# Melanophore Migration and Survival during Zebrafish Adult Pigment Stripe Development Require the Immunoglobulin Superfamily Adhesion Molecule Igsf11

**DOI:** 10.1371/journal.pgen.1002899

**Published:** 2012-08-16

**Authors:** Dae Seok Eom, Shinya Inoue, Larissa B. Patterson, Tiffany N. Gordon, Rebecca Slingwine, Shigeru Kondo, Masakatsu Watanabe, David M. Parichy

**Affiliations:** 1 Department of Biology, University of Washington, Seattle, Washington, United States of America; 2 Laboratory of Pattern Formation, Graduate School of Frontier Biosciences, Osaka University, Osaka University, Japan; Stanford University School of Medicine, United States of America

## Abstract

The zebrafish adult pigment pattern has emerged as a useful model for understanding the development and evolution of adult form as well as pattern-forming mechanisms more generally. In this species, a series of horizontal melanophore stripes arises during the larval-to-adult transformation, but the genetic and cellular bases for stripe formation remain largely unknown. Here, we show that the *seurat* mutant phenotype, consisting of an irregular spotted pattern, arises from lesions in the gene encoding Immunoglobulin superfamily member 11 (Igsf11). We find that Igsf11 is expressed by melanophores and their precursors, and we demonstrate by cell transplantation and genetic rescue that *igsf11* functions autonomously to this lineage in promoting adult stripe development. Further analyses of cell behaviors in vitro, in vivo, and in explant cultures ex vivo demonstrate that Igsf11 mediates adhesive interactions and that mutants for *igsf11* exhibit defects in both the migration and survival of melanophores and their precursors. These findings identify the first in vivo requirements for *igsf11* as well as the first instance of an immunoglobulin superfamily member functioning in pigment cell development and patterning. Our results provide new insights into adult pigment pattern morphogenesis and how cellular interactions mediate pattern formation.

## Author Summary

Vertebrate pigment patterns are stunningly diverse and have been an important model of pattern formation for more than a century. Nevertheless, we still know remarkably little about the genes and cell behaviors that underlie the generation of specific patterns. To elucidate such mechanisms, a large number of pigment pattern mutants have been isolated in the genetically tractable zebrafish. Instead of the normal horizontal stripe pattern, many of these mutants exhibit spots of varying sizes and degrees of organization. Here, we show that one such mutant, *seurat*, named for the 19th century pointillist, George *Seurat*, exhibits lesions in the gene encoding a classical cell adhesion molecule (CAM) of the immunoglobulin superfamily, Igsf11. We find that Igsf11 mediates cell adhesion and promotes the migration and survival of melanophores and their precursors during adult stripe formation. These results are exciting because they are the first time that a CAM has been implicated in pigment pattern formation, despite the long-standing expectation that such molecules might be required to regulate adhesive interactions during these events. These cellular phenotypes further represent the first known in vivo functions for Igsf11 and point to the potential for similar activities amongst the rich diversity of immunoglobulin superfamily members.

## Introduction

Pigment patterns are among the most striking of vertebrate traits and nowhere are these patterns more diverse than in teleost fishes [1]–[4]. In this group, a stunning array of pigment patterns function in predation avoidance, shoaling, and mate choice and are thought to have played important roles in speciation [5]–[8]. Among teleosts, the zebrafish Danio rerio has emerged as a useful model organism for uncovering the genetic and cellular bases of pigment pattern development.

The zebrafish adult pigment pattern comprises a series of dark horizontal stripes that include black melanophores, alternating with lighter “interstripes” that include yellow–orange xanthophores; a third class of pigment cells, the iridescent iridophore occurs in both stripes and interstripes. Development of this pattern occurs during the larval-to-adult transformation between ∼2–4 weeks post-fertilization [9]–[12]. At this time, latent precursor cells of presumptive neural crest origin migrate from peripheral nerves and possibly other locations to the hypodermis, between the epidermis and the myotome, where differentiation occurs and the initially intermingled cells organize into stripes [10], [13].

Mutational analyses have identified several loci that are required for the development of adult melanophores [9], [14]–[17], xanthophores [18], and iridophores [19], [20], and these and other approaches have revealed important roles for cellular interactions, particularly between melanophores and xanthophores, in organizing the adult stripe pattern [21]–[24]. Remarkably, these interactions meet the predictions of Turing models of pattern formation that rely on dynamics driven by processes of reaction diffusion with lateral inhibition [25], [26]. Nevertheless, the molecular mechanisms that drive cellular behaviors during stripe formation have remained obscure.

Of particular interest for understanding the genetic mechanisms and cellular behaviors underlying stripe formation are mutants that retain all three classes of pigment cells while nevertheless developing abnormal adult pigment patterns. To date, two such mutants have been analyzed most extensively. The jaguar mutant exhibits fewer stripes than wild-type fish and, within these stripes, melanophores and xanthophores are intermingled [27]–[29]. The jaguar phenotype arises from mutations in kir7.1, encoding an inwardly rectifying potassium channel, expressed and required by cells of the melanophore lineage [28], [30]. By contrast, the leopard mutant [9], [12], [22], [29] exhibits spots rather than stripes of melanophores, a defect arising from mutations in the gap junction gene, connexin41.8, which is expressed by melanophores and xanthophores [31]. The presumed functions of both gene products raised the possibility that physiological ion fluxes contribute to pattern formation; indeed wild-type, but not jaguar (kir7.1) mutant melanophores depolarize as a result of contacts with xanthophores in vitro [28]. Nevertheless, it has remained unclear to what extent genes classically known to regulate other morphogenetic processes are required specifically during pigment stripe formation.

In this study, we analyze the *seurat* mutant phenotype, consisting of an irregular spotting pattern similar to that of the *leopard* mutant. We chose the *seurat* mutant because, unlike some adult pigment mutants [19], [32], [33], defects are found in both body and fin pigment patterns, suggesting the affected locus may function normally in a core aspect of pattern formation. We show that *seurat* corresponds to *immunoglobulin superfamily member 11* (*igsf11*), encoding a cell surface receptor containing two immunoglobulin-like domains. We find that *igsf11* is expressed by the melanophore lineage, promotes the migration and survival of these cells during adult stripe development, and mediates adhesive interactions in vitro. Our results are the first demonstration of *igsf11* functions in vivo, and, more generally, are the first to implicate a major family of “classical” cell adhesion molecule in adult pigment stripe formation. In turn, these findings set the stage for future investigations into how physiological and morphogenetic mechanisms affecting cell migration and survival interact to generate the adult pigment phenotype of zebrafish and other teleosts.

## Results

### *seurat* requirement for patterning adult melanophores

**Figure 1:**
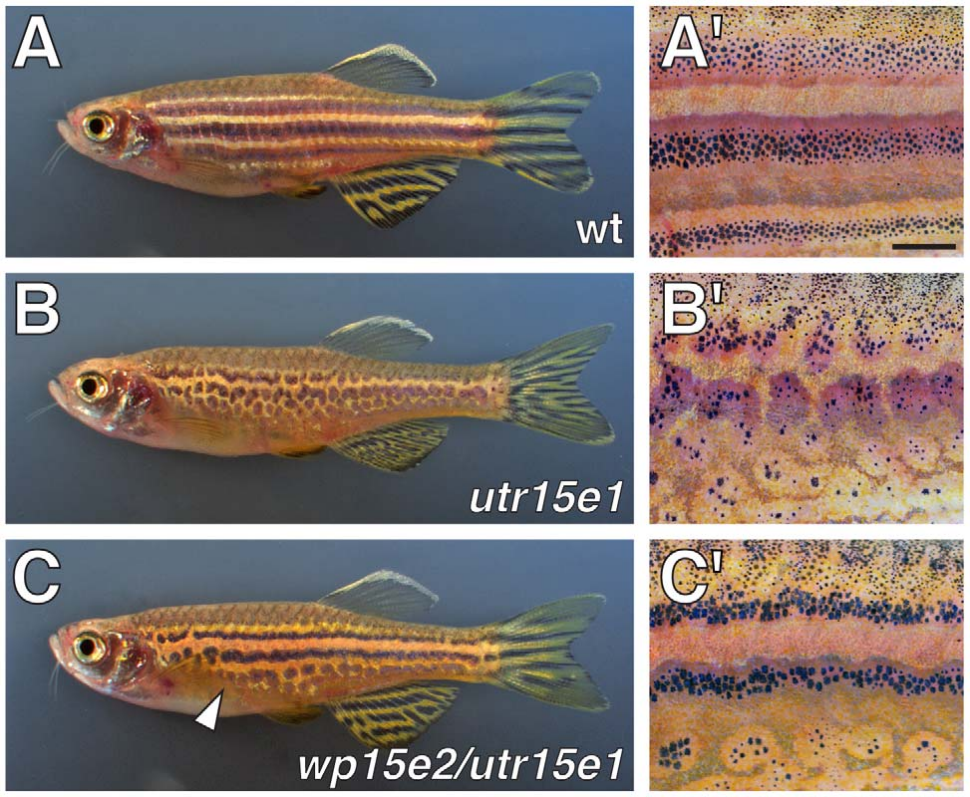
Defective adult pigment stripes in *seurat* mutants. Shown are adult fish (A, B, C) and details of patterns (A′, B′, C′). (A, A′) Wild-type fish exhibit several dark stripes of melanophores and iridophores, as well as light “interstripes” of xanthophores and iridophores. (B, B′) Homozygous *seurat^utr15e1^* mutants exhibit irregular spots of melanophores. (C, C′) *seurat^wp15e2^*/*seurat^utr15e1^* fish exhibit a less severe stripe defect, most evident ventrally (arrowhead). *seurat^wp15e3^*/*seurat^utr15e1^* exhibit a phenotype indistinguishable from *seurat^wp15e2^*/*seurat^utr15e1^*. At this stage, *seurat^wp15e2^* and *seurat^wp15e3^* are nearly indistinguishable from the wild-type when homozygous, though phenotypes are more apparent during the initial stages of stripe formation (not shown). Scale bar: in (A′), 1 mm for (A′–C′).

We isolated the recessive, homozygous viable allele *seurat^utr15e1^* from the inbred AB^wp^ genetic background during a forward genetic screen for ENU-induced mutations affecting adult pigment pattern development. In comparison to the wild-type, *seurat* homozygotes develop fewer adult melanophores, which form irregular spots rather than stripes (Figure 1A, 1B); embryonic and early larval pigment patterns are indistinguishable between wild-type and *seurat* mutants (not shown). We isolated two additional ENU-induced alleles, *seurat^wp15e2^* and *seurat^wp15e3^*, in the wik genetic background by non-complementation screening against *seurat^utr15e1^*. These additional alleles were phenotypically indistinguishable from one another and exhibited less severe phenotypes than *seurat^utr15e1^* (Figure 1C; Figure S1). Gross deficiencies in xanthophore or iridophore numbers were not apparent. For all phenotypic analyses below, we used the stronger allele, *seurat^utr15e1^* (hereafter *seurat*).

### Genetic mosaic analyses reveal a melanophore-autonomous role for *seurat* in stripe development

**Figure 2:**
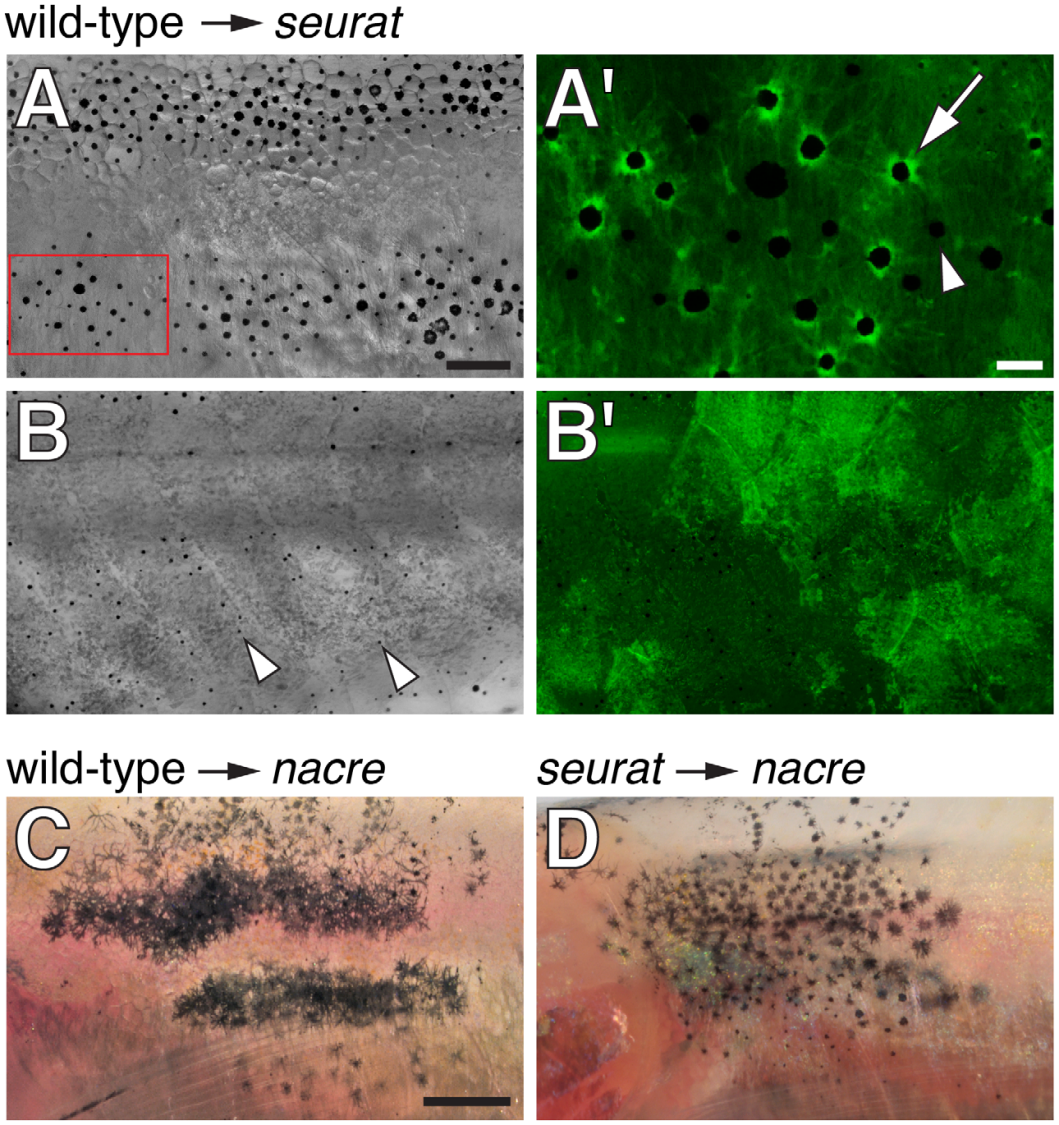
*seurat* is required autonomously to the melanophore lineage. (A, B) Wild-type *Tg(bactin:GFP)* cells transplanted to *seurat* mutant hosts. Fish shown are juveniles (∼13 mm standardized standard length, SSL [11]) and were treated just prior to imaging with epinephrine, which contracts melanosomes towards cell bodies, thereby facilitating the detection of GFP fluorescence. (A) Chimeras that developed wild-type melanophores exhibited patches of restored stripes (*n* = 6). (A′) Detail of boxed region in A, showing GFP+ melanophores (e.g., arrow), as well as occasional GFP−, presumptive *seurat* mutant melanophores (e.g., arrowhead). (B) Chimeras in which wild-type melanophores failed to develop exhibited a *seurat* mutant pattern of dispersed melanophores (arrowheads; *n*>100). In the example shown here, wild-type GFP+ cells developed as epidermis (B′; shown at same magnification as B). (C) When wild-type melanophores differentiated in a *nacre* mutant background, patches of normal stripes developed (*n* = 3; [16]). (D) By contrast, when *seurat* mutant melanophores differentiated in *nacre* hosts, these cells retained a dispersed pattern, as in the *seurat* mutant (*n* = 8), indicating a failure of xanthophores, iridophores, or other cell types to rescue melanophore stripe organization. In additional experiments, in which *nacre; Tg(bactin:GFP)* cells were transplanted to *seurat* mutant hosts, the differentiation of *nacre-* GFP+ (*seurat*+) iridophores likewise failed to rescue melanophore stripes in the *seurat* mutant background (donor xanthophores did not develop in these chimeras; data not shown). Scale bars: in (A) 100 µm for (A,B,B′); in (A′) 20 µm for (A′); in (C) 500 µm for (C,D).

To test if *seurat* acts autonomously to the melanophore lineage in promoting adult pigment stripe formation, we transplanted cells at the blastula stage from phenotypically wild-type *Tg(bactin:GFP)* embryos to homozygous *seurat* mutant embryos and reared the resulting chimeras until adult pigment patterns had formed. If *seurat* acts within the melanophore lineage, we anticipated that wild-type (GFP+) melanophores would form patches more organized than the irregular spots formed by *seurat* mutant melanophores; regions of rescued pattern should include wild-type (GFP+) melanophores but also might include *seurat* mutant (GFP−) melanophores, some of which develop where stripes would normally form (Figure 1B and see below). Consistent with these predictions, we found that wild-type→*seurat* mutant chimeras in which wild-type melanophores developed exhibited large spots or rescued stripes, comprising both wild-type (GFP+) melanophores as well as some *seurat* mutant (GFP−) melanophores (Figure 2). We did not observe these organized patches of melanophores in chimeras that failed to develop wild-type melanophores despite the presence of wild-type epidermis, iridophores, or nerves; we did not observe chimeras that developed donor xanthophores.

To further assess the cell autonomy of *seurat* activities, we transplanted wild-type or *seurat* mutant cells to *nacre^w2^* mutant embryos. nacre mutants fail to develop melanophores owing to a mutation in the *mitfa* transcription factor, which is required autonomously for specifying melanophore fate [15]. Any melanophores developing in these chimeras are thus donor-derived [16]. *nacre* mutants do, however, develop xanthophores and iridophores [27]. If *seurat* acts autonomously to the melanophore lineage, wild-type melanophores should form stripes in the *nacre* mutant background, whereas *seurat* mutant melanophores should fail to do so. Alternatively, if *seurat* effects on melanophore organization are non-autonomous, perhaps acting via xanthophores or another cell type, then both wild-type and *seurat* mutant melanophores should organize into stripes in the *nacre* mutant background [16], [21]. Phenotypes of *seurat* mutant→*nacre* mutant chimeras support an autonomous role for *seurat* within the melanophore lineage, as donor, *seurat* mutant melanophores failed to organize into stripes and instead developed in dispersed patterns, as in the *seurat* mutant (Figure 2C, 2D). Together these data support a model in which *seurat* acts within melanophores or their precursors to promote the organization of these cells into stripes.

### *seurat* corresponds to *immunoglobulin superfamily member 11*

**Figure 3:**
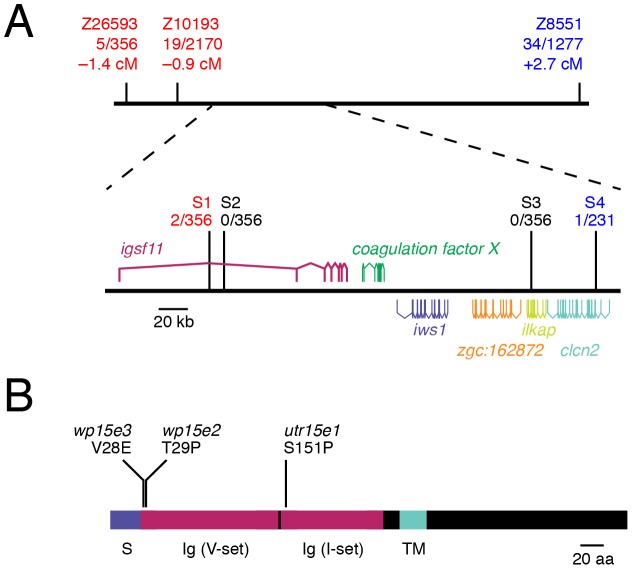
*seurat* mutants exhibit lesions in *igsf11*. (A) Meiotic mapping of the *seurat^utr15e1^* allele revealed a ∼210 kb critical genetic interval harboring several open reading frames of which only *igsf11* exhibits ENU-induced lesions. Differences in numbers of individuals tested across markers reflect the absence of polymorphisms in some mapping families. (B) Schematic of inferred Igsf11 protein showing identified mutations and predicted domains. The lesion in the mapped allele, *seurat^utr15e1^*, occurred in exon 4, whereas lesions in *seurat^wp15e2^* and *seurat^wp15e3^* were found in exon 2. S, predicted signal sequence; TM, predicted transmembrane domain; Ig (V-set), immunoglobulin V-set domain; Ig (I-set), immunoglobulin I-set domain.

To identify the gene affected in *seurat* mutants, we mapped the mutant phenotype to a telomeric region of chromosome 15 between microsatellite markers Z10193 (45.6 Mb) and Z8551 (46.5 Mb) (Figure 3A). Fine-mapping using single nucleotide polymorphisms (S1, S2, S3, S4) within this region revealed a critical genetic interval containing six complete or partial open readings frames. By sequencing exons and cDNAs of each locus and comparing resulting sequences to pre-mutagenized AB^wp^ and wik genetic backgrounds, as well as single nucleotide polymorphisms in the Ensembl database, we identified novel, ENU-induced lesions in *immunoglobulin superfamily member 11 (igsf11; sc:d812)* in each of the three *seurat* alleles (GenBank accession number JQ796184), and found no such lesions in the other candidate genes within this interval. Analyses of the inferred, 442 amino acid Igsf11 peptide sequence revealed a signal sequence, two immunoglobulin-like domains, a transmembrane domain, and a cytoplasmic domain (Figure 3B). The zebrafish peptide sequence exhibited 64% identity and 77% similarity to human IGSF11. In *seurat^utr15e1^* a T→C transition leads to a substitution, S151P, located within the second immunoglobulin domain (Figure 3B; Figure S2). Mutations in the weaker alleles, *seurat^wp15e2^* (T29P) and *seurat^wp15e3^* (V28E) were found at the boundary between the predicted signal sequence and the beginning of the first immunoglobulin domain. These findings suggested that mutations in *igsf11* cause the *seurat* mutant phenotypes.

### *seurat* mutant melanophore patterning is rescued by wild-type *igsf11* expressed in pigment cell lineages

**Figure 4:**
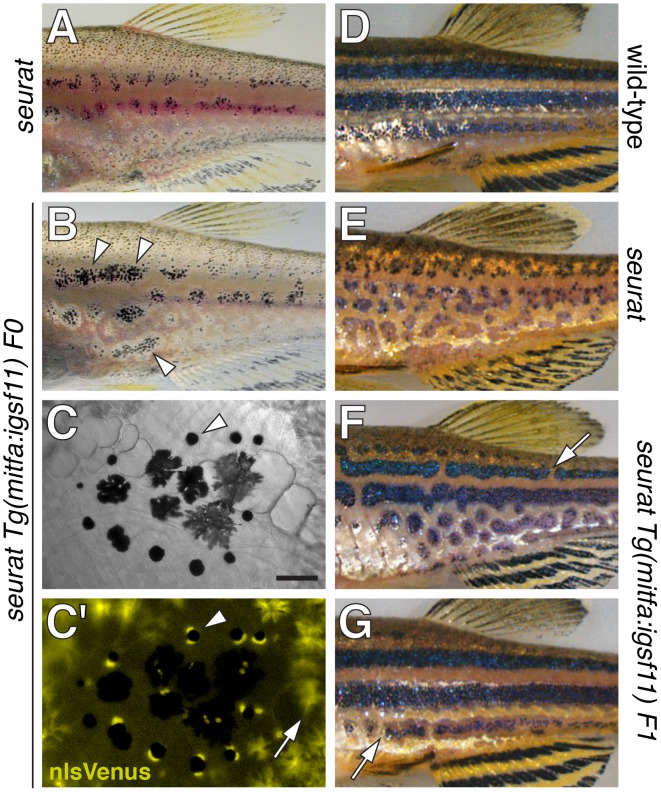
*seurat* mutant phenotype rescued by wild-type *igsf11* expressed in *mitfa*+ pigment cell precursors. (A) Juvenile *seurat* mutant. (B) Sibling of fish in A injected with *mitfa:nlsVenus-V2a-igsf11* transgene at the one cell stage exhibits patches of rescued melanophore stripes (arrowheads). (C,C′) Close-up showing individual melanophores (e.g., arrowhead) that express nlsVenus. This individual had been treated with epinephrine to contract melanin-containing organelles (melanosomes) towards the cell body, which facilitates fluorophore visualization; melanosomes of only some melanophores are in the fully contracted state. Arrow, xanthophores autofluorescence in the same channel as nlsVenus. (D) Adult wild-type. (E) Adult *seurat* mutant. (F, G) Two examples of *seurat* mutants transgenic for stably integrated *mitfa:igsf11*, which exhibit nearly wild-type stripes marked by occasional breaks (arrows). Individuals were genotyped by PCR to verify presence of the transgene. Scale bar: in (C) 60 µm for (C,C′).

To further test the allelism of *igsf11* and *seurat*, we asked if the *seurat* mutant phenotype could be rescued by expressing wild-type *igsf11* cDNA within pigment cells or their precursors. To this end, we constructed transgenes to drive *igsf11* with the *mitfa* promoter [34], [35], which is expressed by precursors to adult melanophores (and possibly iridophores) and newly differentiated melanophores during the larval-to-adult transformation, as well as xanthophores and undifferentiated cells that may be precursors to multiple pigment cell classes in the late larva and adult (Figure S3). We then injected *seurat^utr15e1^* embryos with *mitfa:igsf11* or *mitfa:nlsVenus-V2a-igsf11* transgenes and reared fish through completion of the adult pigment pattern. These genetically mosaic fish expressed nuclear-localizing Venus within the melanophore lineage and exhibited partially rescued stripes (Figure 4A–4C). After screening for germ line carriers, we additionally found that stable transgenic lines expressing *mitfa:igsf11* exhibited stripes nearly indistinguishable from those of the wild-type (Figure 4D–4G). These results and those of positional cloning analyses confirm that seurat corresponds to *igsf11*. In conjunction with the results of cell transplantation analyses, these phenotypes also suggest that *igsf11* promotes normal melanophore stripe development in part by acting through melanophores, or their undifferentiated and possibly multipotent precursors, though we do not exclude the possibility of contributory *igsf11* functions within other lineages as well.

### Expression of *igsf11* by pigment cells, pigment cell precursors, epidermis, and other adult tissues

**Figure 5:**
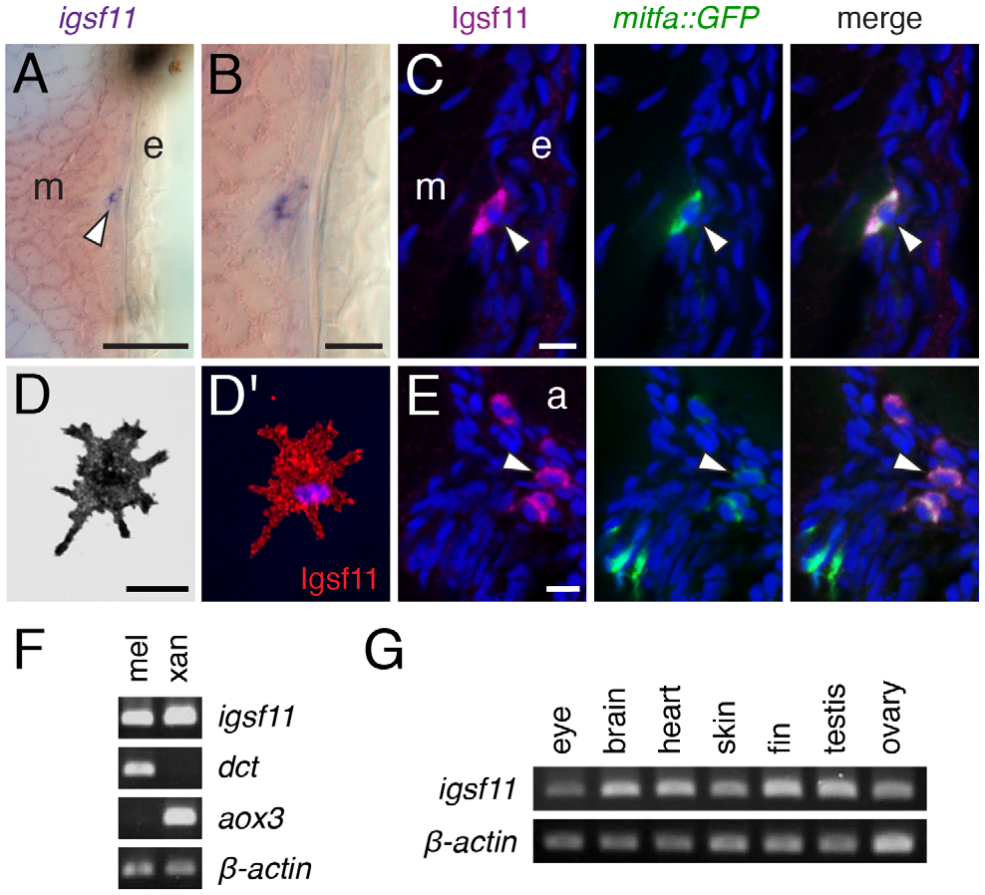
*igsf11* is expressed by pigment cells and their precursors. (A,B) In situ hybridization for *igsf11* transcript during the larval-to-adult transformation, showing an *igfs11*-expressing cell near the hypodermis (A, and higher magnification in B). e, epidermis; m, myotome. (C) Similar location to that shown in (B), illustrating a cell within the hypodermis (arrowhead), coexpressing Igsf11 cell (magenta) and mitfa:GFP (green). Nuclei in all immunofluorescence images are counterstained with DAPI (blue). (D,D′) A melanophore isolated in vitro expresses Igsf11 (red). (E). Extra-hypodermal Igsf11+ cells (arrowhead) also coexpressed Igsf11 (magenta) and mitfa:GFP, though some mitfa:GFP+ cells were Igsf11− (lower left of panels). Shown here are cells just ventral to the aorta (a). (F) RT-PCR showed that cell populations isolated by differential centrifugation and highly enriched for melanophores (mel) and xanthophores (xan) express *igsf11* transcript. *dct*, *dopachrome tautomerase*, expressed by melanophores; *aox3*, *aldehyde oxidase 3*, expressed by xanthophores. β-actin, loading control. (G) *igsf11* expression was detected in several additional tissue types dissected from adult fish. Scale bars: in (A) 40 µm for (A); in (B) 10 µm for (B); in (C) 10 µm for (C,E); in (D) 20 µm for (D).

The above analyses suggest that *igsf11* should be expressed by adult melanophores and perhaps their precursors, though widespread expression in the early embryo [36] suggests the potential for expression more broadly as well. During the larval-to-adult transformation, in situ hybridization revealed *igsf11* transcripts in relatively rare, scattered cells in the hypodermis, where stripe formation takes places between the skin and muscle (Figure 5A, 5B), in extra-hypodermal locations where pigment cell precursors are found [34], and in cells within the spinal cord (Figure S4). A polyclonal antiserum raised against a zebrafish *Igsf11* peptide (Figure S4) revealed an identical distribution of Igsf11-immunoreactive cells. To determine if scattered Igsf11+ cells might be pigment cell precursors, we examined *Tg(mitfa:GFP)^w47^* fish [34], [35]. These analyses revealed that many mitfa:GFP+ cells coexpressed Igsf11 (Figure 5C, 5E), consistent with the autonomous activity of *igsf11* within the pigment cell or melanophore lineages demonstrated by genetic mosaic analyses. Analyses at adult stages further revealed Igsf11 immunoreactivity of isolated melanophores (Figure 5D) and *igsf11* transcripts expressed in isolated cells highly enriched for melanophores and xanthophores (Figure 5F). We did not detect gross differences in levels of Igsf11 immunoreactivity between wild-type and *seurat* mutants, either in sections of larvae or in isolated melanophores, consistent with similar translational efficiency of the wild-type protein and S151P mutant protein (data not shown). Finally, we also detected *igsf11* transcript in several other adult tissues, including the eye, brain, heart, skin, fin, testis and ovary (Figure 5G), presumably reflecting expression by other cells types, or pigment cells or their precursors resident in some of these tissues. Together, RT-PCR, in situ hybridization, and immunohistochemistry support the conclusion that Igsf11 is expressed in adult pigment cells and their precursors in post-embryonic zebrafish.

### Adhesive interactions mediated by Igsf11 *in vitro*

**Figure 6:**
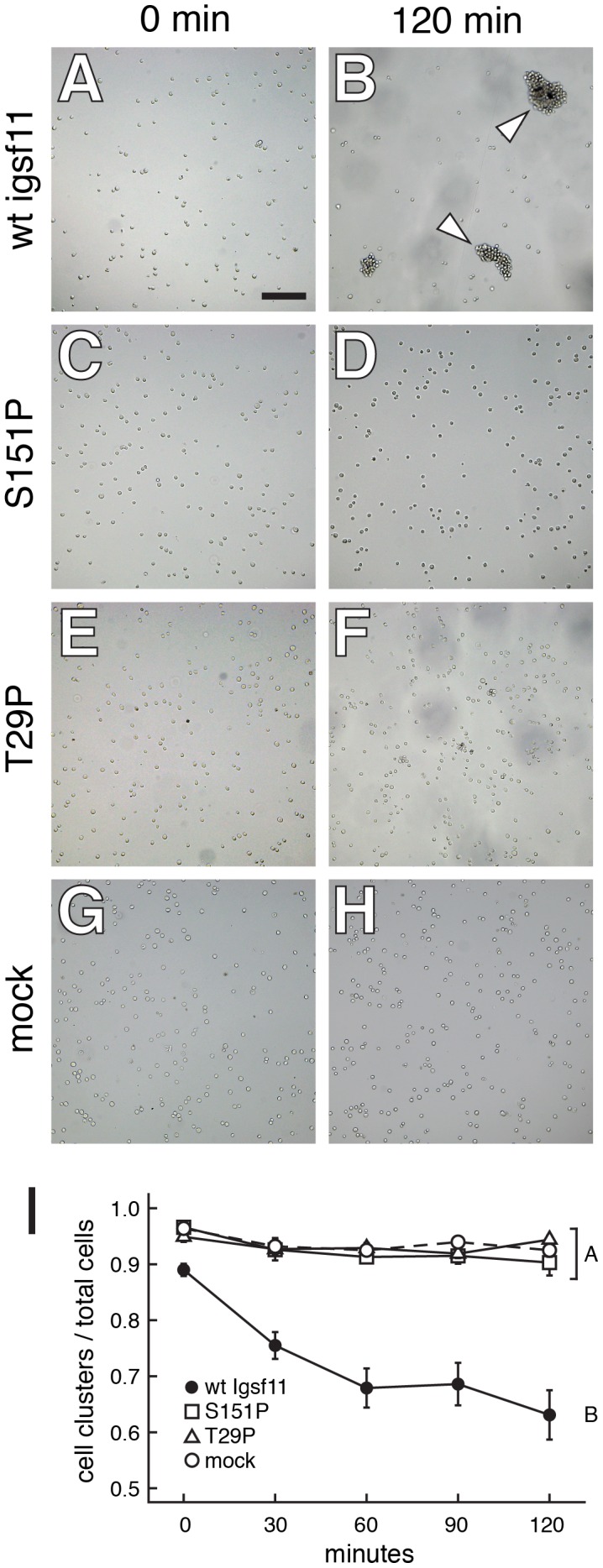
Igsf11 promotes aggregation of K562 myeloid leukemia cells in vitro. Cells were transfected with wild-type zebrafish *igsf11* (A, B), S151P, *seurat^utr15e1^* mutant *igsf11* (C, D), T29P, *seurat^wp15e2^* mutant *igsf11* (E, F), or mock transfected (G, H). At the start of the experiment, the numbers of small cellular aggregates and total numbers of cells were similar (A, C, E, G). By 120 min, a relatively small number of aggregates containing numerous cells had formed in the cells transfected with wild-type *igsf11* (arrowheads in B), though this was not the case in cells of other treatments (D, F, H). (I) Quantitation of the ratio of cellular aggregates to the total number of cells (mean±SE) confirmed that cells were increasingly found in fewer, larger aggregates when transfected with wild-type *igsf11*. At 120 min, degrees of aggregation differed significantly among treatments overall (*F*_3,48_ = 19.8, *P*<0.0001). *Post hoc* means comparisons indicated that aggregation behavior in cells transfected with wild-type *igsf11* (B in the figure) differed significantly from that of cells transfected with mutant *igsf11* or controls (A in the figure; Tukey Kramer post hoc comparisons, *P*<0.01); aggregation of cells transfected with mutant forms of *igsf11* did not differ significantly from one another or from mock transfected cells. Values shown are least squares means adjusted to control for variation after controlling for minor but significant variation among replicates (*P*<0.01).

Immunoglobulin superfamily members mediate a wide range of adhesive interactions. To test if zebrafish Igsf11 also might contribute to adhesive interactions, and the potential of *seurat* mutations to disrupt such interactions, we transfected K562 human myeloid leukemia cells with wild-type or *seurat* mutant *igsf11* cDNAs. In rotary cultures, cells expressing wild-type Igsf11 adhered to one another to form large aggregates within two hours (Figure 6; Figure S5). By contrast, mock transfected cells or cells transfected with S151P (*seurat^utr15e1^*) or T29P (*seurat^wp15e2^*) mutant *igsf11* cDNAs failed to form large aggregates. These findings support a model in which Igsf11 can mediate adhesive interactions *in vivo* and further demonstrate that both mutant forms of Igsf11 are compromised for such activity.

### *igsf11* promotes the migration and survival of melanophores and their precursors

**Figure 7:**
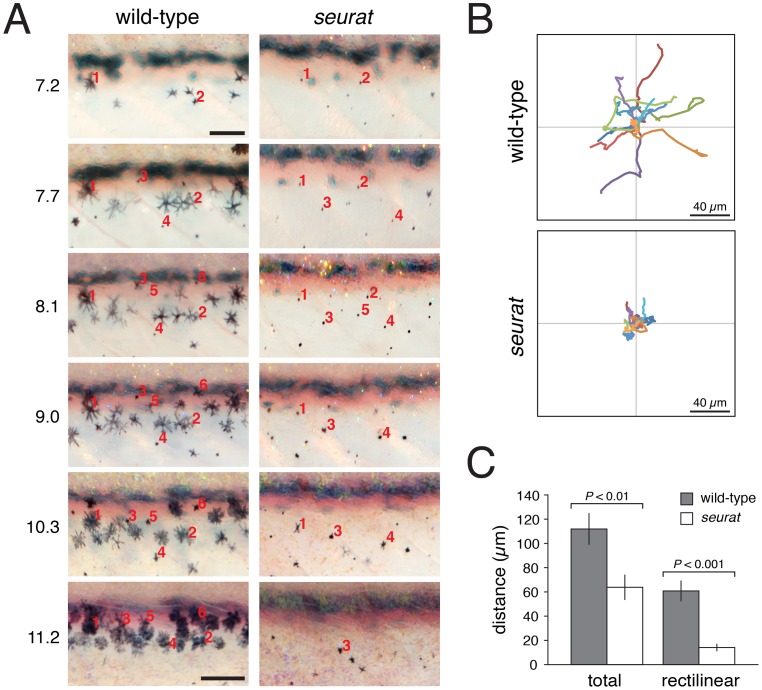
*igsf11*-dependent migration and survival of melanophores. (A) Repeated images of developing wild-type and *seurat* mutant larvae between 14–28 days post-fertilization. Numbers to the left of images are SSL. In wild-type larvae, new adult melanophores differentiated already within stripes or translocated short distances as stripes formed (e.g., note changes in the relative positions of cells 2 vs. 4, and cell 3 vs. 1 and 5). In *seurat* mutants, however, little movement was observed and many melanophores died as evidenced by the presence of melanized cellular debris apparent at high magnification (not shown; [21], [32]. Images shown were rescaled to maintain the same field of view as the fish grew; scale bars at 7.2 SSL and 11.2 SSL represent 100 and 200 µm, respectively. (B) When cultured *in vitro*, wild-type melanophores migrated further than *seurat* mutant melanophores. Shown are tracks of 16 cells of each genotype. (C) Quantification of total and rectilinear distances moved by cells *in vitro* confirmed reduced motility of *seurat* mutant melanophores (*t* = 3.0, *t* = 5.4, respectively; d.f. = 26). Shown are means ± SE.

Our finding that Igsf11 can mediate adhesive interactions in vitro, and the well-known roles of adhesive interactions in promoting cell migration and survival, led us to ask if either of these morphogenetic behaviors were compromised in *seurat* mutants. We repeatedly imaged homozygous *seurat* mutants and heterozygous wild-type siblings through the larval-to-adult transformation. These image series indicated that melanophores in seurat mutants tend to be more punctate than in the wild-type and exhibit reduced rates of migration and an increased likelihood of death as compared to wild-type melanophores (Figure 7A; Videos S1, S2). *seurat* mutants also exhibited a progressively more severe deficiency in melanophore numbers as the larval–to–adult transformation progressed (Figure S6).

To further assess a role for *igsf11* in promoting melanophore migration we compared the motility of wild-type and *igsf11* mutant melanophores in vitro. Similar to phenotypes in vivo, seurat mutant melanophores attached poorly to their substrate resulting in a more rounded appearance, and *seurat* mutant melanophores that did attach migrated significantly shorter distances than wild-type melanophores (Figure 7B, 7C; Videos S3, S4).

**Figure 8:**
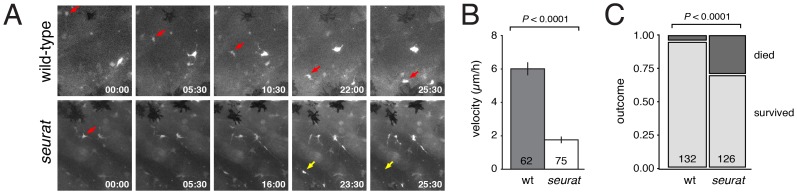
Melanophore precursors require *igsf11* for their migration and survival. (A) Selected frames from time-lapse movies of mitfa:GFP+ cells in wild-type and seurat mutant explants. A single cell (red arrow) moved from dorsal to ventral over the duration of the movie. In a *seurat* mutant, many cells failed to migrate (e.g., red arrow) or died (yellow arrow) during the period of imaging. (B) Velocities (mean±SE) of mitfa:GFP+ cells were significantly reduced in *seurat* mutants compared to the wild-type (*t* = 11.2, d.f. = 135), as were total distances traveled (not shown). (C) *seurat* mutant melanophores were also significantly more likely to die than were wild-type melanophores (*X^2^* = 29.8, d.f. = 1).

Finally, to determine if *igsf11* is required for the migration and survival of melanophore precursors, in addition to differentiated melanophores, we crossed *Tg(mitfa:GFP)^w47^* into the *seurat* mutant background and examined cell behaviors by ex vivo imaging [34]. As for differentiated melanophores, these analyses revealed significantly reduced migration and survival of mitfa:EGFP+ cells in *seurat* mutants as compared to the wild-type (Figure 8, Videos S5, S6, S7, S8).

Together, these analyses demonstrate a requirement for *igsf11* in promoting the migration and survival of melanophores and their precursors, supporting a model in which melanophore organization into stripes is mediated in part through Igsf11-dependent adhesive interactions.

## Discussion

The results of this study identify critical roles for the immunoglobulin superfamily member Igsf11 in the development of zebrafish adult pigment stripes. We found that lesions in *igsf11* are responsible for the *seurat* mutant phenotype of irregular melanophore spots. We demonstrated that Igsf11 promotes adhesive interactions of heterologous cells in vitro, and that *seurat* mutant forms of Igsf11 harboring missense mutations are defective for this activity. By cell transplantation and cell-type specific rescue experiments, we additionally found that *igsf11* acts autonomously to pigment cell lineages in promoting melanophore stripe formation. Finally, our analyses of cellular behaviors in vivo, in vitro, and in ex vivo explants indicate that *igsf11* promotes both the migration and survival of melanophores and their precursors. Whereas roles for cell adhesion molecules in the development of specific pigment patterns have long been suspected [37]–[41], our study is the first to implicate a particular locus expressed by pigment cells in these processes.

Our study expands the known developmental roles of immunoglobulin superfamily (IgSF) proteins, which include such well-studied members as N-CAM, DSCAM and ICAM-1, and provides the first in vivo model system for dissecting the functions of Igsf11 specifically. The immunoglobulin superfamily is an especially diverse set of transmembrane proteins [42], [43], the functions of which have been analyzed most extensively in the nervous system, where they mediate axon guidance and fasciculation, target recognition, and dendrite patterning [44]–[47], as well as in the immune system, where they are required for mediating interactions between immune cells and their environments, and for mounting immune responses [48]–[50]. IgSF members also play important roles in regeneration [51] and in cancer, acting as tumor suppressors or enhancers of invasion [52], [53]. Although IgSF members are not known to be expressed abundantly by normal human melanocytes, several of these genes are dysregulated in melanoma and associated with melanoma progression and metastasis [54], [55] and immunoreactivity using an anti-N-CAM antibody has been detected in xanthophores of some species [56].

*IGSF11* was first identified in mouse and human and shown to be expressed highly in brain and testes (for this reason being named originally Brain- and Testes-specific-IgSF, BT-IgSF) [57]. *IGSF11* was also identified independently as a gene up-regulated frequently in intestinal-type gastric cancers [58]. Our finding that *igsf11* promotes melanophore morphogenesis represents the first identified function for an *igsf11* orthologue in vivo as well as the first evidence of an IgSF member contributing to normal pigment cell development and patterning.

Our finding that *igsf11* is expressed and required by cells of the melanophore lineage and promotes adhesive interactions in vitro suggests two complementary models for the cellular bases of Igsf11-dependent interactions during adult pigment pattern formation. First, Igsf11 could mediate adhesive interactions specifically amongst differentiated melanophores as these cells organize into stripes. Second, Igsf11 could promote stripe development by mediating interactions between melanophores or their possibly multipotent precursors and their environments, either through homophilic or heterophilic adhesive interactions. Our analyses cannot yet speak to the first model, but results of the present study do support the second model of Igsf11-dependent interactions between melanophores and neighboring cell types. For example, we found that mitfa:GFP+ cells exhibited defects in migration and survival in *seurat* mutants, prior to melanization and stripe formation. Likewise, *seurat* mutant melanophores attached poorly to a collagen type IV substrate in serum-containing medium, and exhibited reduced motility independent of interactions with other melanophores.

The biochemical mechanisms for Igsf11-dependent interactions remain unknown. Mammalian IGSF11 mediates homophilic adhesive interactions *in vitro* [59] and such interactions could occur in vivo during adult pigment pattern formation. Yet, our findings that Igsf11 acts autonomously to melanophores or their precursors in cell transplantation and genetic rescue experiments, and that *igsf11* transcripts and protein are not detected in the environment through which these cells migrate, suggest that Igsf11 interacts with one or more heterophilic binding partners to promote melanophore lineage morphogenesis. Indeed, the coxsackie and adenovirus receptor, encoded by CXADR, which is the closest homologue of *IGSF11*, mediates both homophilic and heterophilic adhesive interactions [60]. Consistent with the existence of additional Igsf11 ligands is our observation that different *seurat* mutant forms of Igsf11 equally abrogate cellular aggregation in vitro, despite having either severe or more mild pigment pattern defects in vivo; this outcome suggests that different mutant forms of Igsf11 may be differentially affected in their adhesive interactions with heterologous factors present *in vivo* but not *in vitro*. The existence of other Igsf11 interaction partners also seems likely from our demonstration that isolated wild-type and *seurat* mutant melanophores differed in their motility on Type IV collagen (though we cannot exclude the possibility that IgSF11 could have been present in serum). Future studies aim to elucidate the mechanisms responsible for Igsf11-dependent adhesion, including the identification of *cis-* and *trans-*interacting proteins.

In conclusion, our results identify a new gene required for adult pigment pattern formation and suggest an essential role for Igsf11-dependent adhesive interactions in promoting the morphogenesis of melanophores and their precursors during development of the adult form. It will be especially interesting to learn how pathways dependent on “classical” cell adhesion molecules of the sort identified here interact with physiological mechanisms mediated by kir7.1 and other factors [28] to orchestrate pigment pattern formation in zebrafish and other teleosts.

## Materials and Methods

### Ethics statement

All work in this study was conducted in accordance with guidelines and approved protocols for animal care and use at the University of Washington and Osaka University.

### Isolation of *seurat* mutant alleles

*seurat^wp15e1^* was isolated in a forward genetic, early pressure screen for N-ethyl-N-nitrosourea (ENU) induced mutations in the AB^wp^ genetic background and was subsequently maintained in the same, unmutagenized background. Additional alleles, *seurat^wp15e2^* and *seurat^wp15e2^*, were isolated as ENU-induced mutations in the wik genetic background by screening against *seurat^wp15e1^*, with subsequent backcrosses of non-complementing individuals to confirm allelism of new mutations.

### Cell transplantation

Chimeric embryos were generated by transplanting cells at blastula stages (3.3–3.8 hours post-fertilization) and then rearing through late juvenile stages by which time an adult pattern has formed [11]. The *Tg(bactin:GFP)* transgenic line was provided by Ken Poss.

### Positional cloning and sequence analyses

*seurat* was mapped to chromosome 15 by bulked segregant analyses of fish derived from mapping crosses constructed using seurat (AB^wp^ genetic background) and wik, then subsequently mapped between microsatellite markers Z10193 and Z8551. Additional single nucleotide polymorphisms (S1, S2, S3, S4) were identified within this region of chromosome 15 (45.8∼46.1 Mb) and were used to narrow the critical genetic interval in additional mapping crosses generated in Tubingen and AB genetic backgrounds. Differences in total numbers of individuals tested reflect background-specific polymorphisms and numbers of informative individuals analyzed. Gene predictions were derived from Ensembl (Sanger Institute). cDNA sequences for all genes in the critical interval were compared to those of the un-mutagenized AB^wp^ as well as other backgrounds. To test for lesions that might affect mRNA splicing, exons and flanking intronic sequences of these loci were examined from genomic DNA as well, though the presence of numerous repetitive elements in this telomeric region precluded complete sequencing of some splice junctions. Protein domains were predicted using Pfam, CLC Main Workbench 6.6.1 (CLC bio, Muehital Germany) and SignalP 4.0 [61] and by alignment and structural comparison with the closely related coxsackie and adenovirus receptor [60], [62]. Structures were illustrated using Cn3D (http://www.ncbi.nlm.nih.gov/Structure/CN3D/cn3d.shtml).

### Rescue of *seurat* mutant phenotype

Two different plasmid DNAs were generated for rescue experiments. For one construct, *mitfa:igsf11*, the pT2AL200R150G vector [63] was modified by replacing the *ef1a* promoter with a 1.3 kb fragment of the *mitfa* promoter followed by the *igsf11* coding sequence. The second construct, *mitfa:nlsVenus* *-V2a-igsf11*, was generated using the Gateway Tol2kit pDestTol2pA2 vector [64], and included a 2.2 kb fragment of the mitfa promoter followed by a composite open reading frame generated by overlap extension PCR [65] that consisted of a nuclear-localizing Venus fluorophore and the *igsf11* coding sequence, linked by a V2a peptide breaking sequence to allow the production of separate peptide products [66]. Rescue constructs and *Tol2* mRNA synthesized *in vitro* were injected into homozygous *seurat^utr15e1^* embryos at the one-cell stage. Effects of transgenes were evaluated in these injected, mosaic fish, and in the non-mosaic F1 progeny of germ-line carriers for the *mitfa:igsf11* transgene, in which genomic incorporation of the transgene was verified by PCR.

### RT–PCR analysis of *igsf11* expression

Zebrafish adult tissues were harvested following euthanasia by methyl methane sulfonate (MMS, Sigma) overdose. Total RNAs were obtained using the RNeasy Protect Mini Kit (Qiagen), and cDNA generated with SuperScript III CellsDirect cDNA Synthesis System (Invitrogen). 4.4 ng of the cDNAs (RNA equivalent) obtained from each organ were used in PCRs to detect expression of *igsf11* expression or β-actin as a positive control. PCR amplifications were performed for 32 cycles for *igsf11* and 27 cycles for *β-actin* at 95°C for 30 s, at 60°C for 30 s, and 72°C for 30 s.

To test for *igsf11* expression in adult melanophores and xanthophores, fin pigment cells were isolated and cDNAs synthesized. Zebrafish were anesthetized with MMS and then fin regions containing melanophores and xanthophores, respectively, were dissected under a stereomicroscope. Fin clips were treated with solution containing 2.5 mg/ml trypsin liquid (Worthington), 1.2 mg/ml BSA (Sigma) and 1 mM EDTA (Wako) in PBS for 10 min at 28°C. Trypsin solution was then removed and the tissue rinsed several times with PBS, after which samples were incubated for 60 min at 28°C with solution containing 1 mg/ml collagenase I (Worthington), 0.1 mg/ml DNase I (Worthington), 0.1 mg/ml STI (Worthington), 1.2 mg/ml BSA, and 100 nM epinephrine (Sigma) in PBS. Suspension solutions were filtered with 25 µm mesh, followed by density-gradient centrifugation at 30× g for 15 min at room temperature in 50% Percoll (Sigma), precipitating separate populations of melanophores and xanthophores to ∼95% purity from other contaminating cell types such as epidermis. To evaluate cross-contamination of melanophores and xanthophores, we tested for expression of dct and aox3, which are specifically expressed by melanophores and xanthophores, respectively [18], [67]. PCR amplifications were performed for 40 cycles for *igsf11*, 34 cycles for *dct* and *aox3*, and 38 cycles for *β-actin* at 95°C for 30 s, at 60°C for 30 s, and 72°C for 30 s. Primer sets were designed to span introns. For *igsf11*: 5′-TCTGATCGCGGGCACCATCG-3′, 5′-TAGGTGTTGGTGGACGTCAGAGTG-3′; *β-actin*: 5′-CGGTTTTGCTGGAGATGATG-3′, 5′-CGTGCTCAATGGGGTATTTG-3′; *dct*: 5′-ATCAGCCCGCGTTCACGGTT-3′, 5′-ACACCGAGGTGTCCAGCTCTCC-3′; *aox3*: 5′-AGGGCATTGGAGAACCCCCAGT-3′ and 5′-ACACGTTGATGGCCCACGGT-3′.

### Immunohistochemistry and in situ hybridization

Polyclonal antisera for zebrafish Igsf11 were generated in mouse. The peptide immunogen selected, PTYAWEKQESVPKLPHN, occurs within the second predicted immunoglobulin domain of Igsf11. This antiserum did not recognize a specific fragment of the predicted size in Western blots, therefore its specificity was assessed by injecting embryos at the one-cell stage with a morpholino oligonucleotide (Gene Tools, LLC) targeting the *igsf11* translational start site (igsf11-MO: CATGTTTCCCAGCGAAAGTCGTCGT) to test for reduced immunoreactivity at 24 hours post-fertilization. Morpholino was injected at 2 ng or 4 ng per embryo, as determined by an absence of toxicity at these doses using a 5 base pair mismatch control morpholino (igsf11-MM: CATcTTTgCCAcCGAAAcTCcTCGT). Antiserum was used at 1∶500–1∶1000 following fixation in 4% paraformaldehyde and detected using goat anti-mouse Alexa 555 or Alexa 568 secondary antibodies. For immunohistochemistry of larvae, individuals of 7.0–9.0 standardized standard length (SSL; [11]) were fixed in 4% paraformaldehyde containing 1% DMSO in PBS, embedded in OCT, and sectioned by cryostat at 18–20 µm. In situ hybridization on vibratome sections followed [17] using a full length (1329 bp) *igsf11* cDNA for synthesis of antisense and sense riboprobes.

### Aggregation assay

The human myeloid leukemia cell line (K562) was maintained with RPMI-1640 medium (Sigma) containing 10% FBS (Invitrogen). pIRES2-igsf11 plasmid for nucleofection, a modified electroporation technique, was generated by cloning of full length Igsf11 fragment into pIRES2-AcGFP (Clontech). Cells transiently expressing Igsf11 proteins (wild-type or mutant) were obtained using a 4D-Nucleofector (Lonza) with pIRES2-igsf11 plasmid. Twenty four hours after the nucleofection, cultures were suspended into single-cells by repeated pipetting, centrifuged and then resuspended in HBSS (Gibco) containing 1 mM CaCl_2_ at a density of 6×10^4^ cells/ml. 500 µl of cell suspensions were transferred into a 24-well culture plate. The plate was then rotated on a gyratory shaker (80 rpm) at 37°C for 2 h. Three independent experiments were performed and 5 random fields of view for cells in each treatment were imaged every 30 min. The degree of adhesion was evaluated as the ratio of the number of cell clusters over the total number of cells. Data were analyzed for effects of treatment and replicate by analyses of variance (ANOVA) in JMP 8.0.2 (SAS Institute, Cary NC) after *arc sin* transformation to control for heteroscedasticity of residuals that is common for ratio data [68]. Differences between specific treatments were assessed by Tukey Kramer *post hoc* comparisons. In additional experiments, transfected cells were split after centrifugation, with half of each sample used for aggregation assays and the other half used either for verifying the equivalence of transfection efficiencies of wild-type and mutant *igsf11* constructs by fluorescence activated cell sorting (BD FACS Calibur, BD Biosciences) for GFP expression (5000 cells per sample). Similar levels of wild-type and mutant Igsf11 protein expression were also examined by immunocytochemistry.

### Repeated imaging and melanophore counts of fish during larval-to-adult transformation

Larvae were viewed and imaged with an Olympus SZX-12 stereomicroscope and Axiocam HR camera. For time-course analyses, individual fish from a *seurat*/+ backcross were imaged daily and genotypes determined retrospectively. Fish were reared individually and imaged after brief anesthetization with MMS. Complete image series were obtained for 5 wild-type and 8 *seurat* mutant individuals. For determination of melanophore numbers, all melanophores were counted between the dorsal and ventral margins of the flank in a region bounded by the anterior margin of the dorsal fin and the posterior margin of the anal fin. Counts were obtained from individual larvae at selected standardized standard lengths during the larval-to-adult transformation with genotypes and binned sizes analyzed as fixed effects in analyses of variance. For depicting pattern development in animations shown in Videos S1 and S2, all images were aligned and rescaled to control for growth using Adobe Photoshop CS5.

### Analyses of melanophore motility *in vitro*

Melanophores were isolated from adult fish as described above and then re-suspended in L15 (Sigma) without FBS. Cells were cultured in 96-well culture dishes that had been coated with type IV collagen (BD Biosciences). After one overnight incubation at 28°C, culture medium was changed with fresh L15 containing 5% FBS and the cells were imaged using an Olympus IX71 microscope equipped with an Olympus DP72 digital camera and Lumina Vision software (Mitani Corporation).

Melanophores that attached, survived at least 48 h, and did not interact with other melanophores were chosen for analysis. Melanophore centroid positions were obtained in ImageJ (http://rsb.info.nih.gov/ij/) and plotted every hour between 12–36 h after medium change. Rectilinear migration distance was defined as the length between the beginning and ending positions for each cell.

### Analyses of motility and survival *ex vivo*

To image the morphogenetic behaviors of presumptive melanophore precursors ex vivo, 7.0 SSL larvae were rinsed with 10% Hanks medium and then anesthetized and then decapitated with a razor blade. Larval trunks were then placed on 0.4 µM transwell membranes (Milipore) in glass bottom dishes containing L15 medium, 3% fetal bovine serum, and penicillin/streptomycin. The trunks were equilibrated for 3 h at 28.5°C then imaged at 30 min intervals for 18–26 h on a Zeiss Observer inverted epifluorescence microscope with an Axiocam MRm camera. Z-stacks of 10–15 planes collected at 4 µm intervals were merged for final analyses.

## Supporting Information

Figure S1 Developmental phenotype of weak allele, *seurat^wp15e2^*. (A) Wild-type juvenile. (B) Homozygous *seurat^wp15e2^* juvenile. Arrow, typical irregularity in melanophore stripe border. Arrowhead, break in melanophore stripe. Scale bar: in (A) 5 mm for (A,B).
(TIF)

Figure S2 Comparison of *seurat* lesions in zebrafish Igsf11 to structural predictions for mammalian coxsackie and adenovirus receptor. (A) Alignment of amino acid sequences showing locations of *seurat* mutations in zebrafish Igsf11 relative to mouse coxsackie and adenovirus receptor (accession: 3JZ7_A). Boxed, S151P (*utr15e1*). Line above, V28E and T29P (*wp15e3* and *wp15e2*, respectively). (B) Mapping of V28E, T29P and S151P lesions onto crystal structure 3MJ7_B, representing the coxsackie and adenovirus receptor (in complex with the junctional adhesion molecule-like protein, JAML, not shown) [62]. Homologous residues to V28E and T29P are predicted to be immediately N-terminal to the first immunoglobulin domain as shown here, or within the N-terminal region of the first (V-set) immunoglobulin-like domain by Pfam (see Figure 3). The residue homologous to S151P is located within the second immunoglobulin domain of the mouse protein. (C) Mapping of S151P onto crystal structure 3JZ7, representing a homomeric dimer of the coxsackie and adenovirus receptor (both subunits shown) [60]. Residues homologous to V28E and T29P were not included in the 3JZ7_A structure despite their occurrence in the 3JZ7_A sequence shown in A.
(TIF)

Figure S3 mCherry expression driven by the *mitfa* promoter. Shown is expression from a 1.3 kb fragment of the *mitfa* promoter in mosaic, transiently transgenic late larval fish (∼9.5 SSL). (A) mitfa:mCherry was expressed by newly differentiated melanophores (arrow) as well as undifferentiated cells that may be precursors to melanophores and xanthophores (arrowhead). (B) Expression of mitfa:mCherry in differentiated xanthophores, which autofluoresce in the GFP channel (arrow), as well as in undifferentiated cells (arrowhead). Expression from a 2.2 kb fragment of the *mitfa* promoter was similar to that shown here.
(TIF)

Figure S4 Characterization of Igsf11 antiserum. (A) Knockdown by morpholino oligonucleotide injection reduced Igsf11 immunoreactivity in embryos at 24 hours post-fertilization. Igsf11 immunoreactivity was present along vertical myosepta in uninjected embryos as well as embryos injected with a control igsf11 5 bp mismatch morpholino (igsf11-MM), but was dramatically reduced in embryos injected with a morpholino targeting the igsf11 translational start site (igsf11-MO). Embryos were injected with 4 ng of either morpholino and exposure times were identical for all images shown. (B,C) In addition to scattered cells in the hypodermis and extra-hypodermal locations (main text), both in situ hybridization (B) and immunohistochemistry (C) revealed igsf11-expressing cells (arrowheads) in the spinal cord during the larval-to-adult transformation (larvae shown here at ∼9 SSL [11]). Staining appears more extensive in B than C owing to different section thicknesses (150 µm, 20 µm, respectively).
(TIF)

Figure S5 Transfection efficiency and expression of wild-type and mutant Igsf11 by K562 human myeloid leukemia cells. (A) Fluorescence activated cell sorting indicated similar transfection efficiencies for cells transfected with wild-type or mutant forms of Igsf11. (B) Immuncytochemistry confirmed expression of wild-type and mutant forms of Igsf11 by K562 cells (shown here without rotary culturing or aggregration). Mock treated cells were transfected with pIRES2-AcGFP1vector alone.
(TIF)

Figure S6 An adult melanophore deficiency in *seurat* mutants. *seurat* mutants exhibit an increasingly severe melanophore deficiency as adult pigment pattern formation progresses (genotype, *F*_1,29_ = 5.2, *P*<0.05; genotype x size class interaction, *F*_3,29_ = 96.6, *P*<0.0001), with a significant difference in melanophore numbers relative to wild-type emerging by late stages of adult pigment pattern formation as assessed by Tukey-Kramer *post hoc* comparisons of means. Numbers of embryonic melanophores at 5 days post-fertilization were indisinguishable between wild-type and *seurat* mutant early larvae, both in the dorsal stripe (*F*_1,18_ = 0.7, *P* = 0.4) and the lateral stripe (*F*_1,18_ = 0.01, *P* = 0.9).
(TIF)

Video S1 Development of wild-type pigment pattern. Animation over ∼2 weeks shows the gradual accumulation of new melanophores and progressively more organized stripes with only infrequent loss of melanophores. Individual images were rescaled to control for growth allowing behaviors of individual cells to be more clearly apparent.
(MOV)

Video S2 Development of *seurat* mutant pigment pattern. Shown is a homozygous mutant sibling of the wild-type fish in Video S1. In contrast to the wild-type, melanophores tend to be punctate, migrate little and are frequently lost.
(MOV)

Video S3 Behaviors of wild-type melanophores *in vitro*. These cells were typically well spread and highly motile. The melanophore outlined in red is an example of a cell for which movements were tracked and quantified over the 48 h duration of the movie.
(MOV)

Video S4 Behaviors of *seurat* mutant melanophores *in vitro*. In contrrast to the wild-type, *seurat* mutant melanophores were often poorly spread and largely failed to migrate.
(MOV)

Video S5 Behavior of pigment cell precursors in wild type. Overview of wild-type trunk imaged *ex vivo* showing extensive migration of mitfa:GFP+ cells.
(MOV)

Video S6 Behavior of pigment cell precursors in wild type. Detail of Video S5 showing a migrating mitfa:GFP+ cell traversing from dorsal to ventral (red arrow) as well as a rare mitfa:GFP+ cell undergoing fragmentation (yellow arrow).
(MOV)

Video S7 Behavior of pigment cell precursors in *seurat* mutant. Overview of *seurat* mutant trunk, showing similar numbers of mitfa:GFP+ cells to that observed in the wild-type (Video S5) but reduced motility and increased frequency of death amongst these cells.
(MOV)

Video S8 Behavior of pigment cell precursors in *seurat* mutant. Detail of Video S7 showing a mitfa:GFP+ cell that failed to migrate (red arrow), as well as three mitfa:GFP+ cells that were successively lost (yellow arrows). Death of cells continuing to express GFP were revealed by their fragmentation followed by rapid, presumably macrophage-dependent, clearance of cellular debris. This fragmentation and rapid disappearance of cells was quite distinct from the gradual changes that result from live cells migrating to different focal planes [34].
(MOV)
