# Supplementary figures and images for: Melanophore Migration and Survival during Zebrafish Adult Pigment Stripe Development Require the Immunoglobulin Superfamily Adhesion Molecule Igsf11
Source: PLoS Genet. 2012 Aug 16;8(8):e1002899. doi: 10.1371/journal.pgen.1002899 (PMC3420941; doi:10.1371/journal.pgen.1002899)

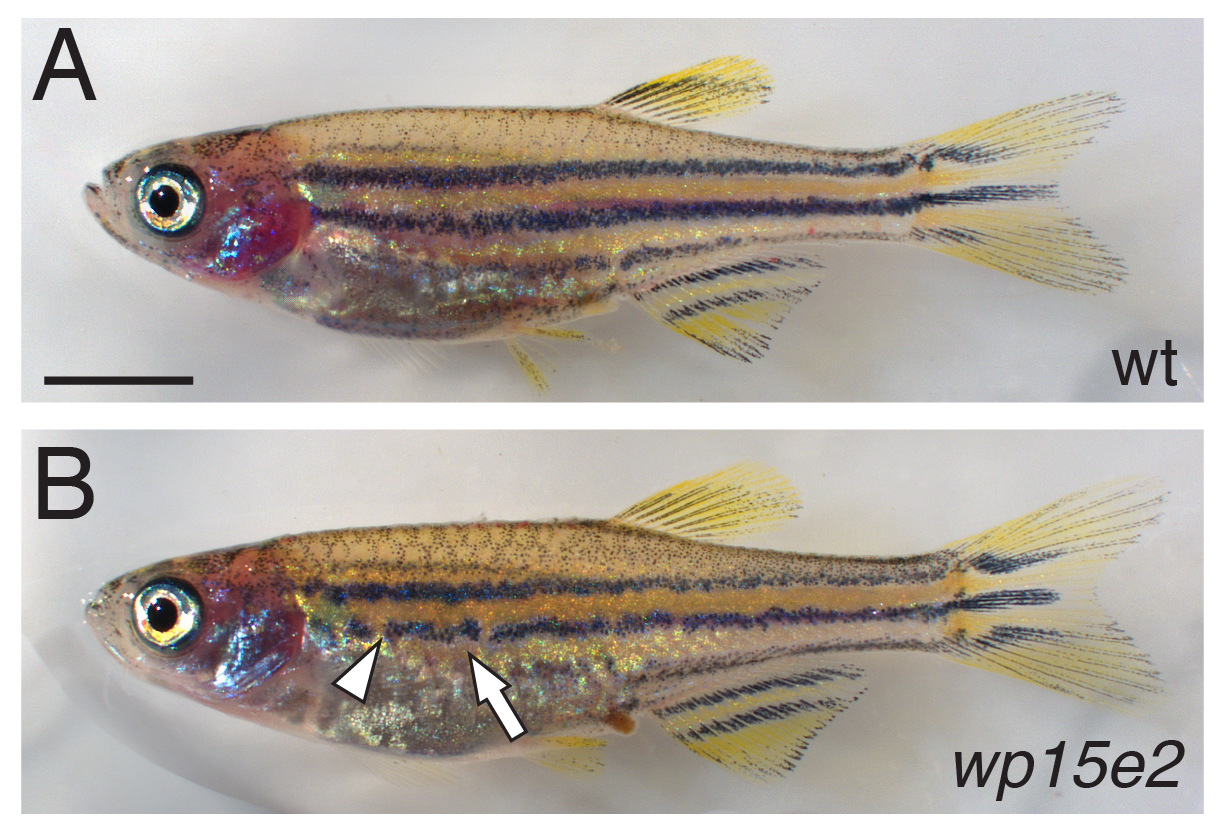

Supplement: Figure S1 — Developmental phenotype of weak allele, seuratwp15e2. (A) Wild-type juvenile. (B) Homozygous seuratwp15e2 juvenile. Arrow, typical irregularity in melanophore stripe border. Arrowhead, break in melanophore stripe. Scale bar: in (A) 5 mm for (A,B). (TIF) [file pgen.1002899.s001.tif]

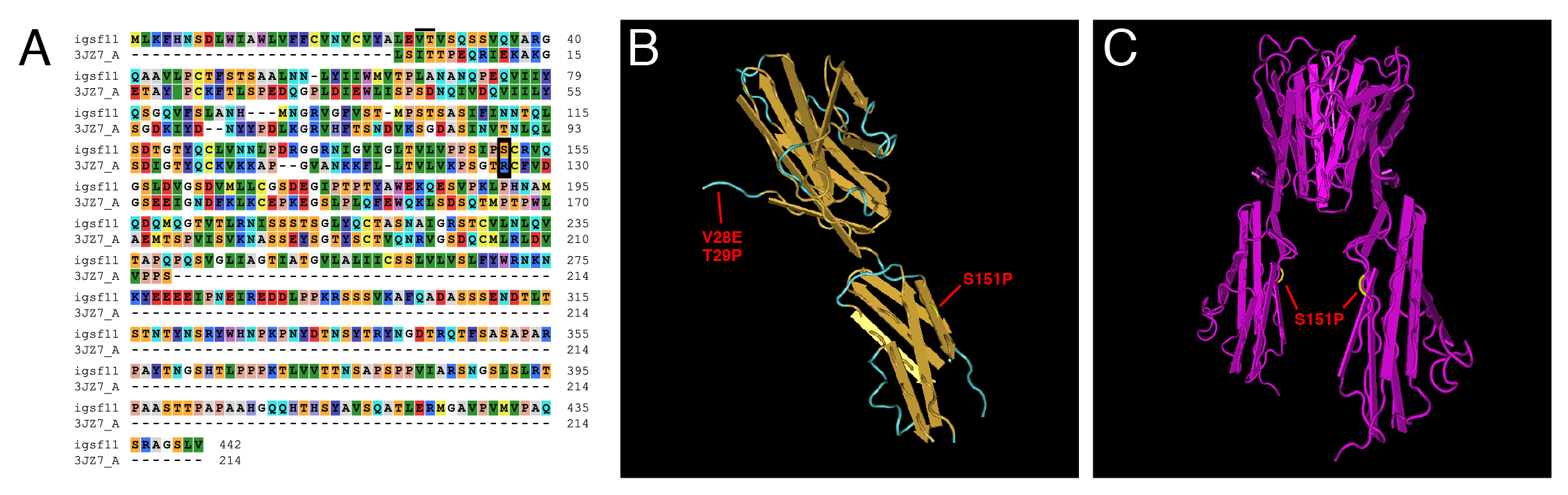

Supplement: Figure S2 — Comparison of seurat lesions in zebrafish Igsf11 to structural predictions for mammalian coxsackie and adenovirus receptor. (A) Alignment of amino acid sequences showing locations of seurat mutations in zebrafish Igsf11 relative to mouse coxsackie and adenovirus receptor (accession: 3JZ7_A). Boxed, S151P (utr15e1). Line above, V28E and T29P (wp15e3 and wp15e2, respectively). (B) Mapping of V28E, T29P and S151P lesions onto crystal structure 3MJ7_B, representing the coxsackie and adenovirus receptor (in complex with the junctional adhesion molecule-like protein, JAML, not shown) [62]. Homologous residues to V28E and T29P are predicted to be immediately N-terminal to the first immunoglobulin domain as shown here, or within the N-terminal region of the first (V-set) immunoglobulin-like domain by Pfam (see Figure 3). The residue homologous to S151P is located within the second immunoglobulin domain of the mouse protein. (C) Mapping of S151P onto crystal structure 3JZ7, representing a homomeric dimer of the coxsackie and adenovirus receptor (both subunits shown) [60]. Residues homologous to V28E and T29P were not included in the 3JZ7_A structure despite their occurrence in the 3JZ7_A sequence shown in A. (TIF) [file pgen.1002899.s002.tif]

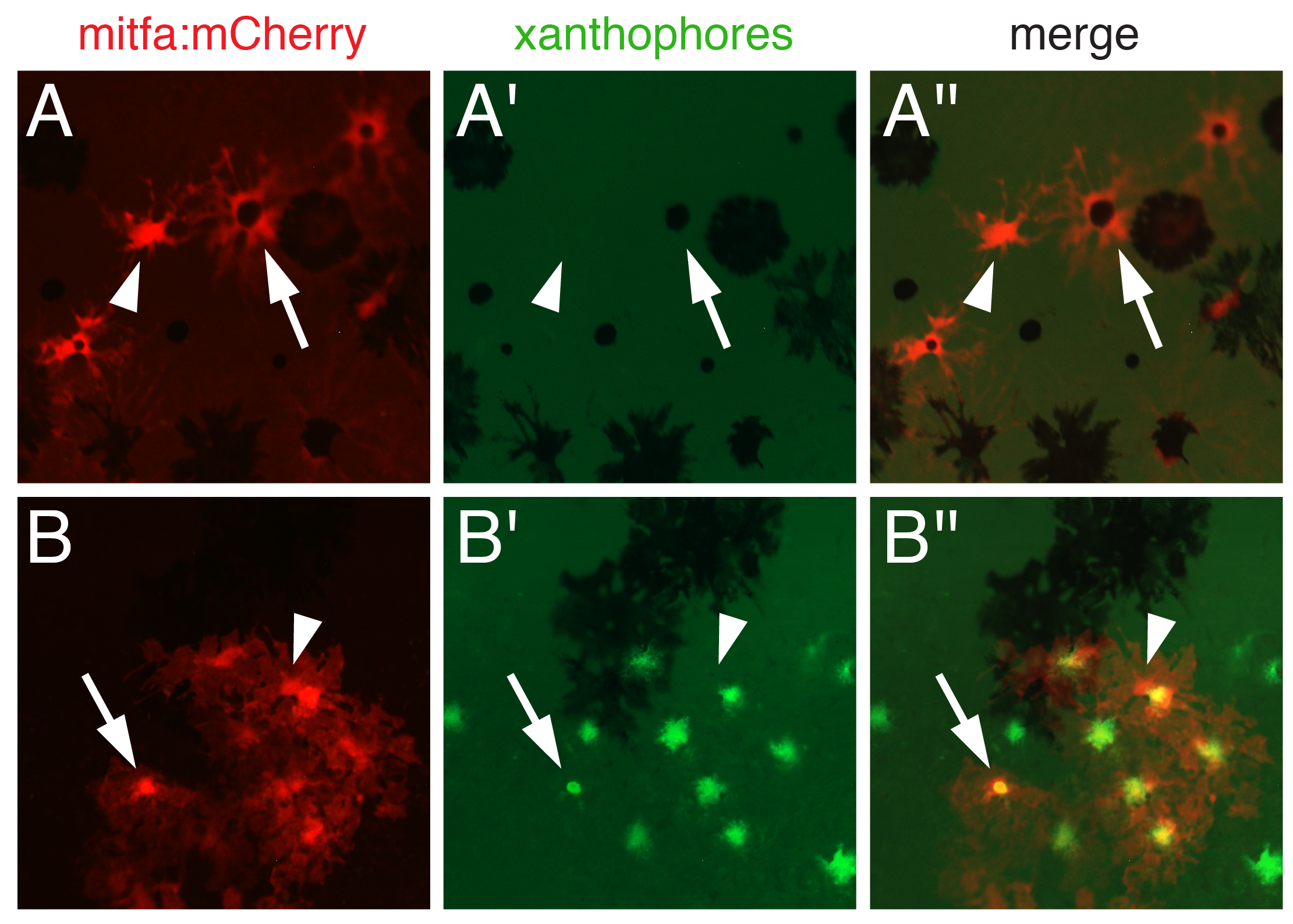

Supplement: Figure S3 — mCherry expression driven by the mitfa promoter. Shown is expression from a 1.3 kb fragment of the mitfa promoter in mosaic, transiently transgenic late larval fish (∼9.5 SSL). (A) mitfa:mCherry was expressed by newly differentiated melanophores (arrow) as well as undifferentiated cells that may be precursors to melanophores and xanthophores (arrowhead). (B) Expression of mitfa:mCherry in differentiated xanthophores, which autofluoresce in the GFP channel (arrow), as well as in undifferentiated cells (arrowhead). Expression from a 2.2 kb fragment of the mitfa promoter was similar to that shown here. (TIF) [file pgen.1002899.s003.tif]

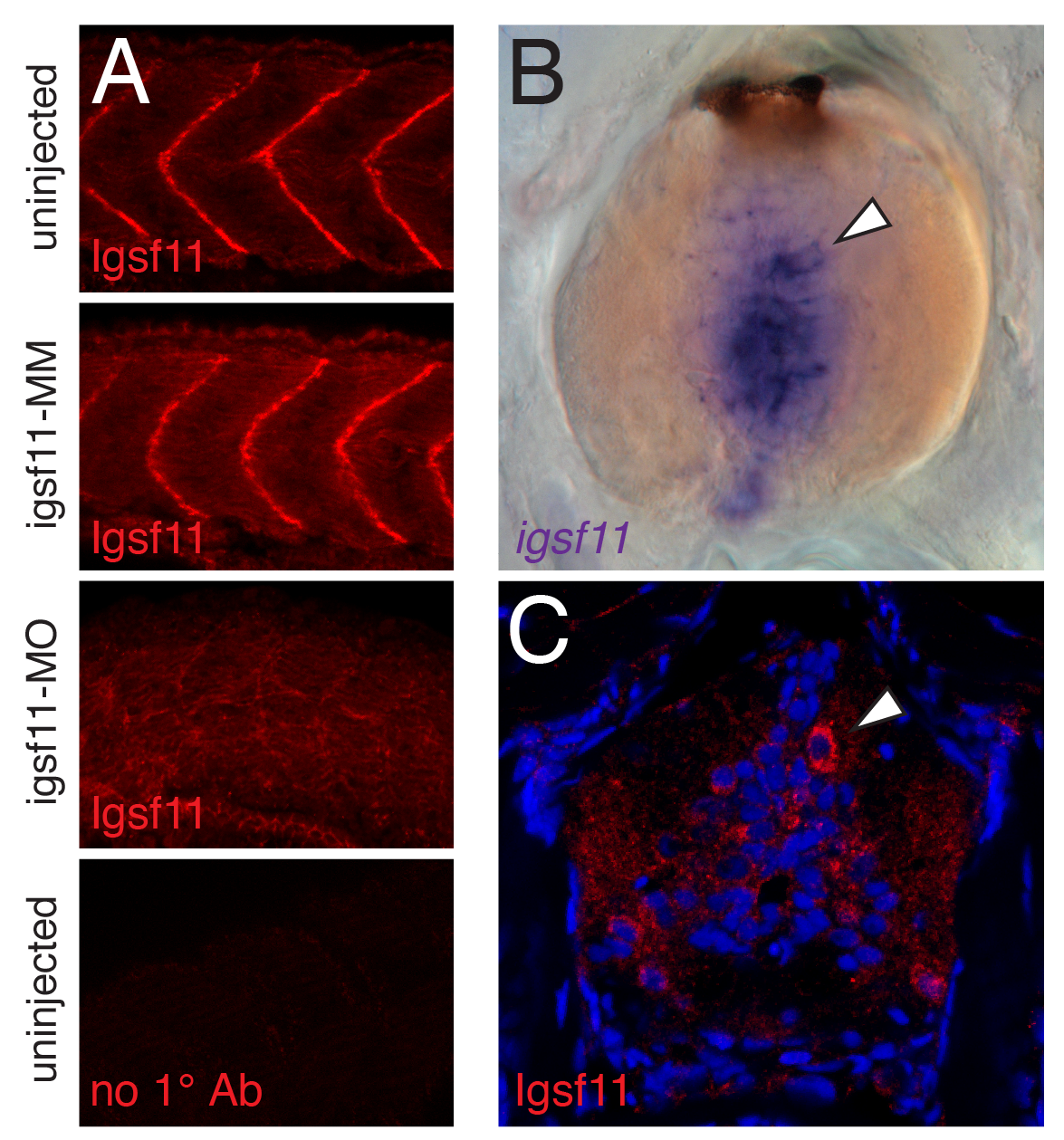

Supplement: Figure S4 — Characterization of Igsf11 antiserum. (A) Knockdown by morpholino oligonucleotide injection reduced Igsf11 immunoreactivity in embryos at 24 hours post-fertilization. Igsf11 immunoreactivity was present along vertical myosepta in uninjected embryos as well as embryos injected with a control igsf11 5 bp mismatch morpholino (igsf11-MM), but was dramatically reduced in embryos injected with a morpholino targeting the igsf11 translational start site (igsf11-MO). Embryos were injected with 4 ng of either morpholino and exposure times were identical for all images shown. (B,C) In addition to scattered cells in the hypodermis and extra-hypodermal locations (main text), both in situ hybridization (B) and immunohistochemistry (C) revealed igsf11-expressing cells (arrowheads) in the spinal cord during the larval-to-adult transformation (larvae shown here at ∼9 SSL [11]). Staining appears more extensive in B than C owing to different section thicknesses (150 µm, 20 µm, respectively). (TIF) [file pgen.1002899.s004.tif]

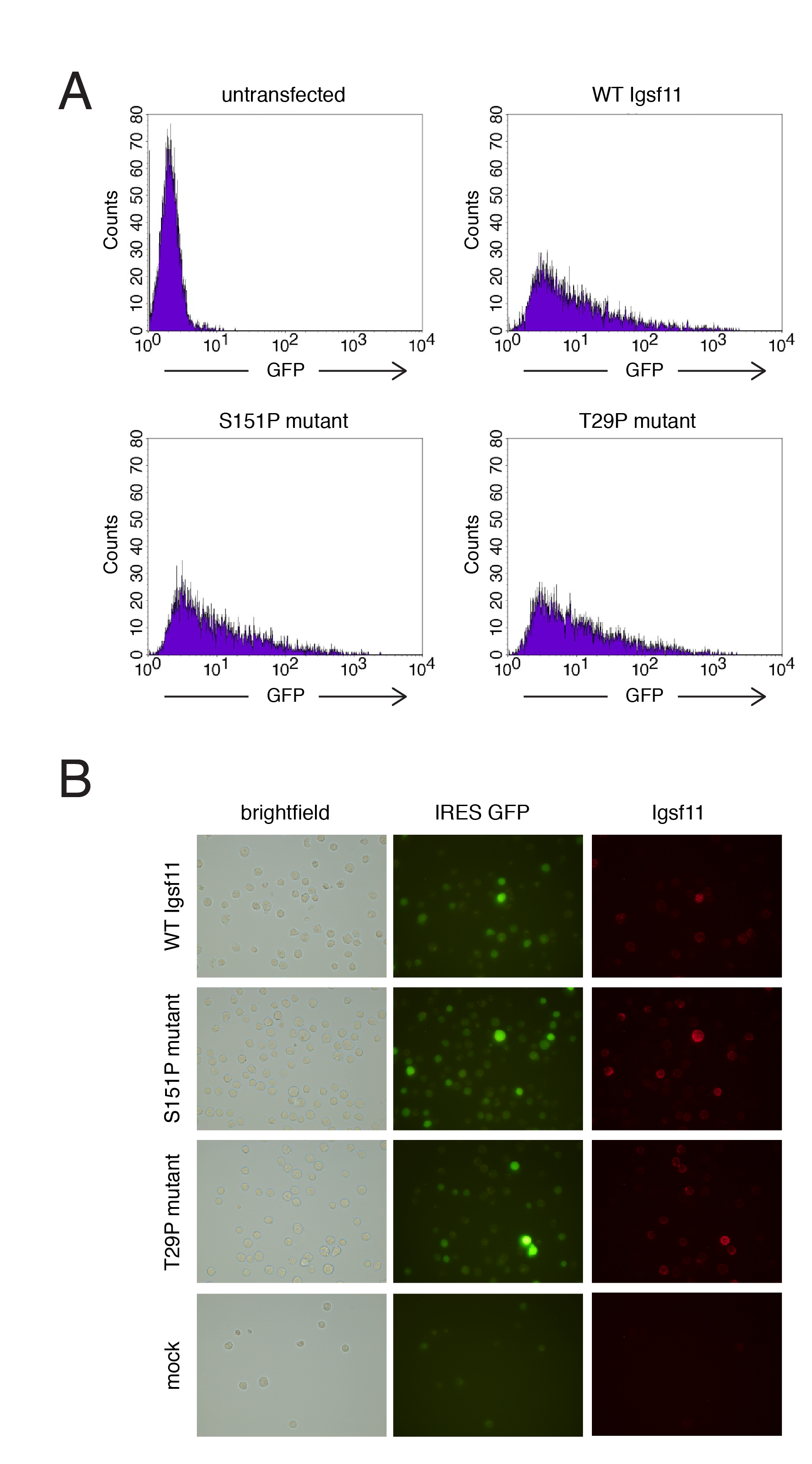

Supplement: Figure S5 — Transfection efficiency and expression of wild-type and mutant Igsf11 by K562 human myeloid leukemia cells. (A) Fluorescence activated cell sorting indicated similar transfection efficiencies for cells transfected with wild-type or mutant forms of Igsf11. (B) Immuncytochemistry confirmed expression of wild-type and mutant forms of Igsf11 by K562 cells (shown here without rotary culturing or aggregration). Mock treated cells were transfected with pIRES2-AcGFP1vector alone. (TIF) [file pgen.1002899.s005.tif]

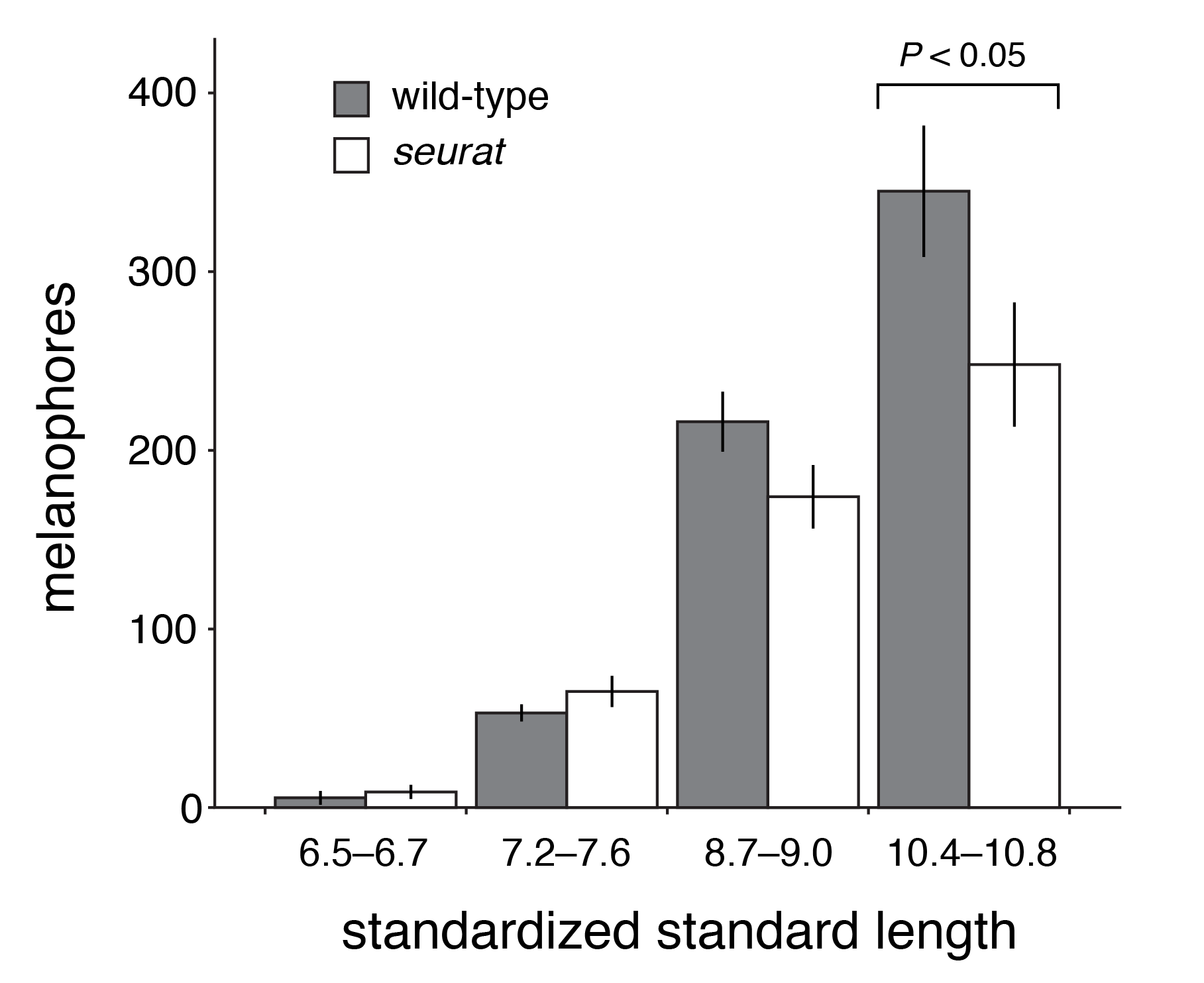

Supplement: Figure S6 — An adult melanophore deficiency in seurat mutants. seurat mutants exhibit an increasingly severe melanophore deficiency as adult pigment pattern formation progresses (genotype, F 1,29 = 5.2, P<0.05; genotype x size class interaction, F 3,29 = 96.6, P<0.0001), with a significant difference in melanophore numbers relative to wild-type emerging by late stages of adult pigment pattern formation as assessed by Tukey-Kramer post hoc comparisons of means. Numbers of embryonic melanophores at 5 days post-fertilization were indisinguishable between wild-type and seurat mutant early larvae, both in the dorsal stripe (F 1,18 = 0.7, P = 0.4) and the lateral stripe (F 1,18 = 0.01, P = 0.9). (TIF) [file pgen.1002899.s006.tif]
